# Genetic diversity and population structure of pigeonpea (*Cajanus cajan* [L.] Millspaugh) landraces grown in Benin revealed by Genotyping-By-Sequencing

**DOI:** 10.1371/journal.pone.0271565

**Published:** 2022-07-20

**Authors:** Géofroy Kinhoégbè, Gustave Djèdatin, Rachit Kumar Saxena, Anu Chitikineni, Prasad Bajaj, Johiruddin Molla, Clément Agbangla, Alexandre Dansi, Rajeev Kumar Varshney

**Affiliations:** 1 Laboratory of Molecular Biology and Bioinformatics Applied to Genomics, National University of Sciences, Technologies Engineering and Mathematics of Abomey, Dassa-Zoumé, Benin; 2 Centre of Excellence in Genomics and Systems Biology, International Crop Research Institute for the Semi-Arid Tropics, Hyderabad, India; 3 Laboratory of Molecular Genetic and Genomes Analysis, University of Abomey-Calavi, Abomey-Calavi, Benin; 4 Laboratory of Biotechnology, Genetic Resources and Plant and Animal Breeding, National University of Sciences Technologies Engineering and Mathematics of Abomey, Dassa-Zoumé, Benin; Faculty of Agriculture (FoA), Sher-e-Kashmir University of Agricultural Sciences and Technology of Kashmir (SKUAST-K), Wadura Campus, INDIA

## Abstract

Genetic diversity studies provide important details on target trait availability and its variability, for the success of breeding programs. In this study, GBS approach was used to reveal a new structuration of genetic diversity and population structure of pigeonpea in Benin. We used a total of 688 high-quality Single Nucleotide Polymorphism markers for a total of 44 pigeonpea genotypes. The distribution of SNP markers on the 11 chromosomes ranged from 14 on chromosome 5 to 133 on chromosome 2. The Polymorphism Information Content and gene diversity values were 0.30 and 0.34 respectively. The analysis of population structure revealed four clear subpopulations. The Weighted Neighbor Joining tree agreed with structure analyses by grouping the 44 genotypes into four clusters. The PCoA revealed that genotypes from subpopulations 1, 2 and 3 intermixed among themselves. The Analysis of Molecular Variance showed 7% of the total variation among genotypes while the rest of variation (93%) was within genotypes from subpopulations indicating a high gene exchange (Nm = 7.13) and low genetic differentiation (PhiPT = 0.07) between subpopulations. Subpopulation 2 presented the highest mean values of number of different alleles (Na = 1.57), number of loci with private alleles (Pa = 0.11) and the percentage of polymorphic loci (P = 57.12%). We discuss our findings and demonstrate how the genetic diversity and the population structure of this specie can be used through the Genome Wide Association Studies and Marker-Assisted Selection to enhance genetic gain in pigeonpea breeding programs in Benin.

## Introduction

Pigeonpea (*Cajanus cajan* [L.] Millspaugh) is an important food legume crop widely grown in tropical and subtropical climates [1]. It is a diploid (2n = 22) crop with a genome size of 833.07 Mbp [2]. Pigeonpea is counted among the five comestible legumes used for food, fodder and firewood [3]. It constitutes a rich source of protein and is used to supplement cereals [4, 5].

In Benin, pigeonpea is largely cultivated and consumed in Adja socio-linguistics area in the South-East of the country and in some others socio-linguistics areas where it improves household income [6, 7]. Despite the importance of this legume, till date, no research effort has been directed towards pigeonpea crop improvement in the country. In general, there are several factors that contribute to the current level situation of pigeonpea development in the country. This includes lack of sufficient characterization of genetic resources and therefore, lack of genetic data [8].

The success of breeding programs, in any crop, depends on the extent of genetic diversity through different traits available to breeders for breeding desirable varieties [9]. These parameters significantly affect parents’ choice for the hybridization and the adopted selection procedures [10]. In addition, by showing the differences which exist among accessions [11], the genetic diversity studies provide important details on target trait availability and its variability for the success of breeding programs [12–14]. Thus, before establishing conservation and improvement programs of pigeonpea genetic resources in Benin, it is necessary to discover the level of genetic diversity and population structure of cultivated landraces.

Different methods are usually used to access genetic diversity. One of them is the agro-morphological traits approach, given the accessibility of their easily measurable characteristics is the most used approach to establish relationships between genotypes and provide information for varieties improvement programs [15, 16]. Hence, agro-morphological traits have been utilized in pigeonpea germplasm’s characterization of both world reference collections [17] and national collections [18–23]. In Benin, agro-morphological traits have already been used in the evaluation of pigeonpea genetic diversity [24–26]. However, the environmental conditions affect agro-morphological traits. Hence, morphological characters do note only reflect the genetic constitution of the cultivars but also the available genetic diversity [27].

Molecular markers are one of the powerful tools used in the characterization of genetic resources [28]. In recent years, the advancement in pigeonpea genomic resources is a result of the development of molecular markers and genome sequence data which are all essentials in molecular breeding approach [29]. Thus, in several crops, a few approaches have been used for simultaneous SNP discovery and genotyping such as Restriction site Associated DNA sequencing (RADseq) [30] and Genotyping-By-Sequencing (GBS) [31], etc. Among these approaches, GBS is the simplest and most cost-effective approach [31] and has proven itself in trait mapping and Genome-Wide Association Studies (GWAS) as well as in diversity studies [32] in various crops like chickpea [33], common bean [34], wheat [35], pigeonpea [36], etc.

In Benin, SNPs markers have been used to assess genetic diversity and population structure of cultivated pigeonpea [37]. However recently, new morphotypes of cultivated pigeonpea have been identified in the country and corresponding accessions collected [7]. This assumes a new structure of the pigeonpea diversity other than that reported by the previous study [37].

Therefore, in the present study, GBS approach was used to reveal a new structure within the pigeonpea genetic resource and to provide important information for pigeonpea breeding programs. Consequently, the objective of the present study was to characterize the genetic diversity and population structure of 50 pigeonpea landraces collected in Benin. We hypothesized that beninese pigeonpea germplasm encompasses more genetic diversity than reported by Zavinon et al. [37] who stated that there are three genetic groups and three subpopulations using SNPs markers.

## Materials and methods

### Plant materials

The material included 50 pigeonpea landraces collected in Benin and held in the genebank of the Laboratory of Molecular Biology and Bioinformatics applied to Genomics of the National University of Sciences, Technologies Engineering and Mathematics of Abomey in Benin and at International Crop Research Institute for the Semi-Arid Tropics at Patancheru in India. These genotypes are originated from 39 villages (S1 Table).

### Germplasm and DNA isolation

Total DNA was isolated from two to three young leaves using a high-throughput DNA extraction protocol. The quality of the DNA was checked on 0.8% agarose gels. DNA concentration was assessed with NanoDrop ND-1000 (Thermo Fisher Scientific Inc., Waltham, USA) and extract purity using the absorbance ratio 260nm to 230nm. According to the DNA concentration, different dilution rates were applied to each sample to normalize each sample at 10 ng/μl for further use.

### Library construction and Genotyping by Sequencing (GBS)

GBS libraries were prepared following Elshire et al. [31] and in accordance with the genomic analysis platform of the Center of Excellence in Genomics and Systems Biology at International Crops Research Institute for the Semi-Arid Tropics in India. Thus, the restriction enzyme ApeKI was used to digest each sample. The digested product was ligated to adapters with cohesive ends (GWC) by addition of T4 DNA ligase enzyme [36] before being incubated at 22°C and heated at 65°C for 1h and 30 min respectively to make the T4 DNA ligase enzyme inactivate. The digested and ligated products from each sample were mixed in equal proportion to construct the GBS libraries. Each library was then amplified and purified. The libraries were sequenced on an Illumina sequencer HiSeq 2500 platform (Illumina Inc, San Diego, CA, USA).

### SNP calling and genotyping

The raw sequence data generated was filtered using Tassel v5.2.63 [38] analysis pipeline according Saxena et al. [36]. In this manner, SNPs were identified and high-quality SNP genotyping dataset was compiled. The draft genome sequence of pigeonpea described by Varshney et al. [2] was used as a reference to align the compiled dataset using Bowtie 2 software [39]. Subsequently, the alignment file was processed through GBS analysis pipeline for SNP calling and genotyping. Eventually, quality control filters were applied to both SNPs and samples following Zhang et al. [40] and Hussain et al. [41] based on the following criteria. Firstly, variants should be bi-allelic SNPs. Secondly, SNPs with more than 20% of missing information were excluded and lastly the SNP markers with minor allele frequency greater than or equal to 0.05 were retained.

### Statistical analysis

Major Allele Frequency (MAF), gene diversity, and Polymorphic Information Content (PIC) values for all SNP markers, were calculated using Power Marker v.3.25 software [42].

The Structure v2.3.4 Software [43] was used for population structure analysis. The membership of each accession was run for value of K = 1 to K = 10 with the admixture model and correlated allele frequency. For each K, it was replicated 3 times. Each run was implemented with a burn-in period of 50,000 steps followed by 100,000 Monte Carlo Markov Chain replicates. “Structure harvester” (http://taylor0.biology.ucla.edu/structureHarvester/) available online was used to calculate the final population structure. The likelihood value Ln (K) (mean + SD) method [44] was used. This method translates the mean of estimated Ln (Prob of data) values as a function of the probable number of subpopulations (K). It assumes that when K approaches the true value, Ln (K) (mean + SD) reaches a plateau or has an inconsequential variance [44]. Genotypes with membership probabilities greater than or equal to 0.7 were grouped together genotypes with membership probability lower than 0.7 were assigned to the admixture group. Genetic variation within the subpopulations was assed using the fixation index (Fst) and average distances between individuals in same cluster using the Expected Heterozygosity (HE).

To assess genetic diversity, genetic distances across genotypes were calculated using Tassel v5.2.63 [38]. The dissimilarity matrix generated was used to construct Neighbor Joining tree. In addition, Principle Coordinate Analysis (PCoA) and Analysis of Molecular Variance (AMOVA) was performed, based on the Structure results and the differentiation genetic index (PhiPT and Nm) allowed to describe the genetic diversity between and among subpopulations and their degrees of differentiation. Also, the genetic diversity index such as mean values of number of loci with private alleles (Pa), number of different alleles (Na); Shannon’s diversity index (H) and the Shannon’s Information Index (I) were calculated. PCoA and AMOVA performed using GenAlEx 6.503 [45] and SNP data was numerically coded as suggested in GenAlEx user manual.

## Results

### Sequence data and SNP discovery

**Fig 1:**
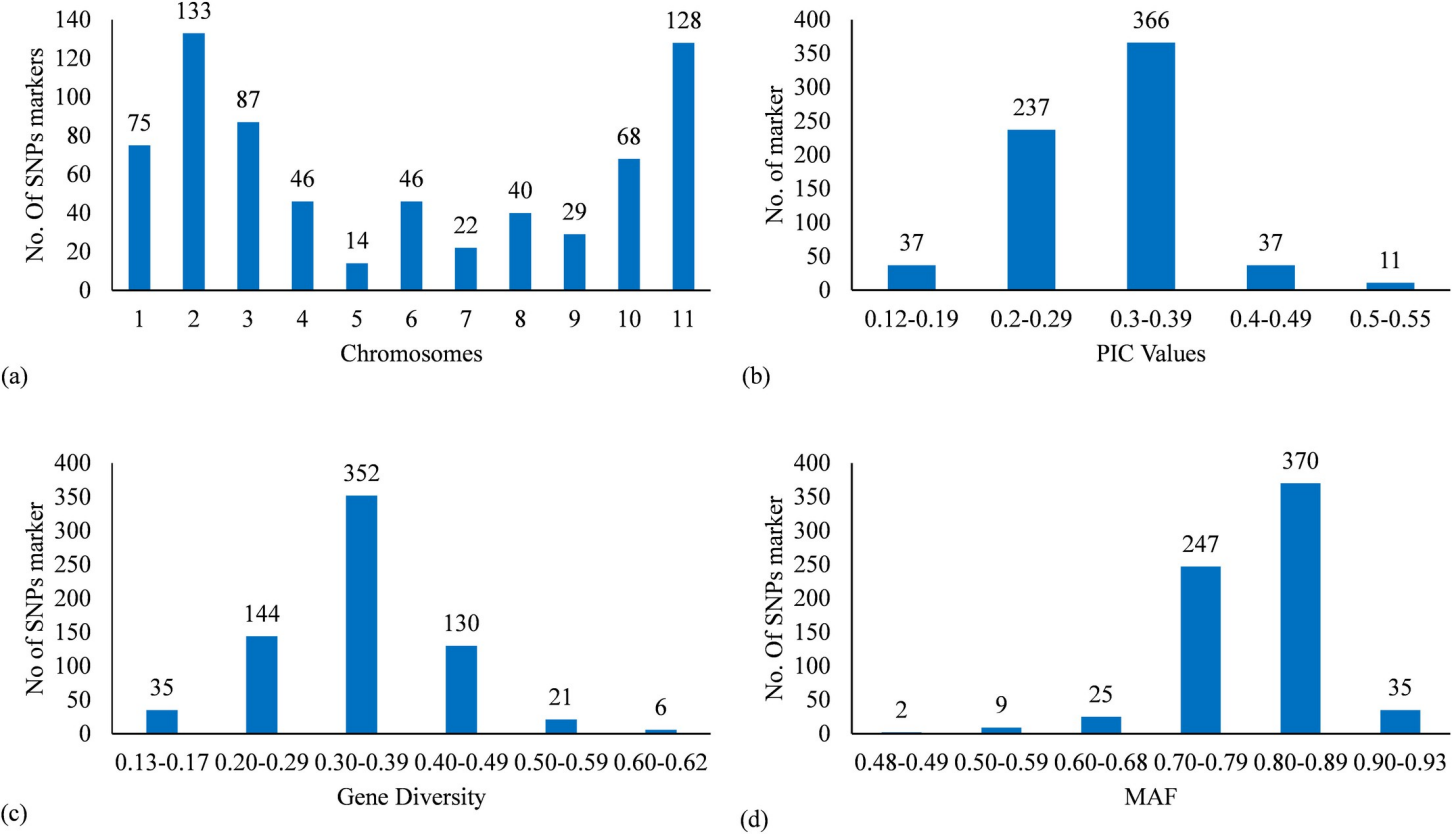
SNPs markers characteristics. (a) Distribution of SNPs across the 11 chromosomes. (b) Polymorphic Information Content (PIC). (c) Variation of Gene Diversity. (d) Frequency distribution of Major Allele Frequency.

Genotyping by sequencing (GBS) of the 50 genotypes provided 18.7 Gb data containing 3.16 million sequence reads. The sequencing data was mapped on to the reference pigeonpea genome and the alignment sequences provided 71,712 SNPs across 50 genotypes. Considering the filtering criteria applied both to samples and SNP, six genotypes (kk7, kk8, kk19, kk29, kk35 and kk36) were excluded from further analysis and a total of 688 high quality SNPs were retained for further analysis. The number of SNPs per chromosome ranged from 14 on chromosome 5 to 133 on chromosome 2 (Fig 1A and S2 Table). The rate of polymorphism was 100% across the other 44 genotypes. The PIC varied from 0.12 to 0.55 with a mean value of 0.30 (Fig 1B). The gene diversity ranged from 0.13 to 0.62 with an average of 0.34 (Fig 1C). A total of 1,376 alleles could be identified. The Major Allele Frequency for all 688 SNPs ranged from 0.48 to 0.93 with an average of 0.80 (Fig 1D). About 2% of SNPs were found to be least informative with PIC and gene diversity values equaling 0.12 and 0.13 respectively and the Major Allele Frequency of 0.93.

### Population structure in pigeonpea landraces

**Fig 2:**
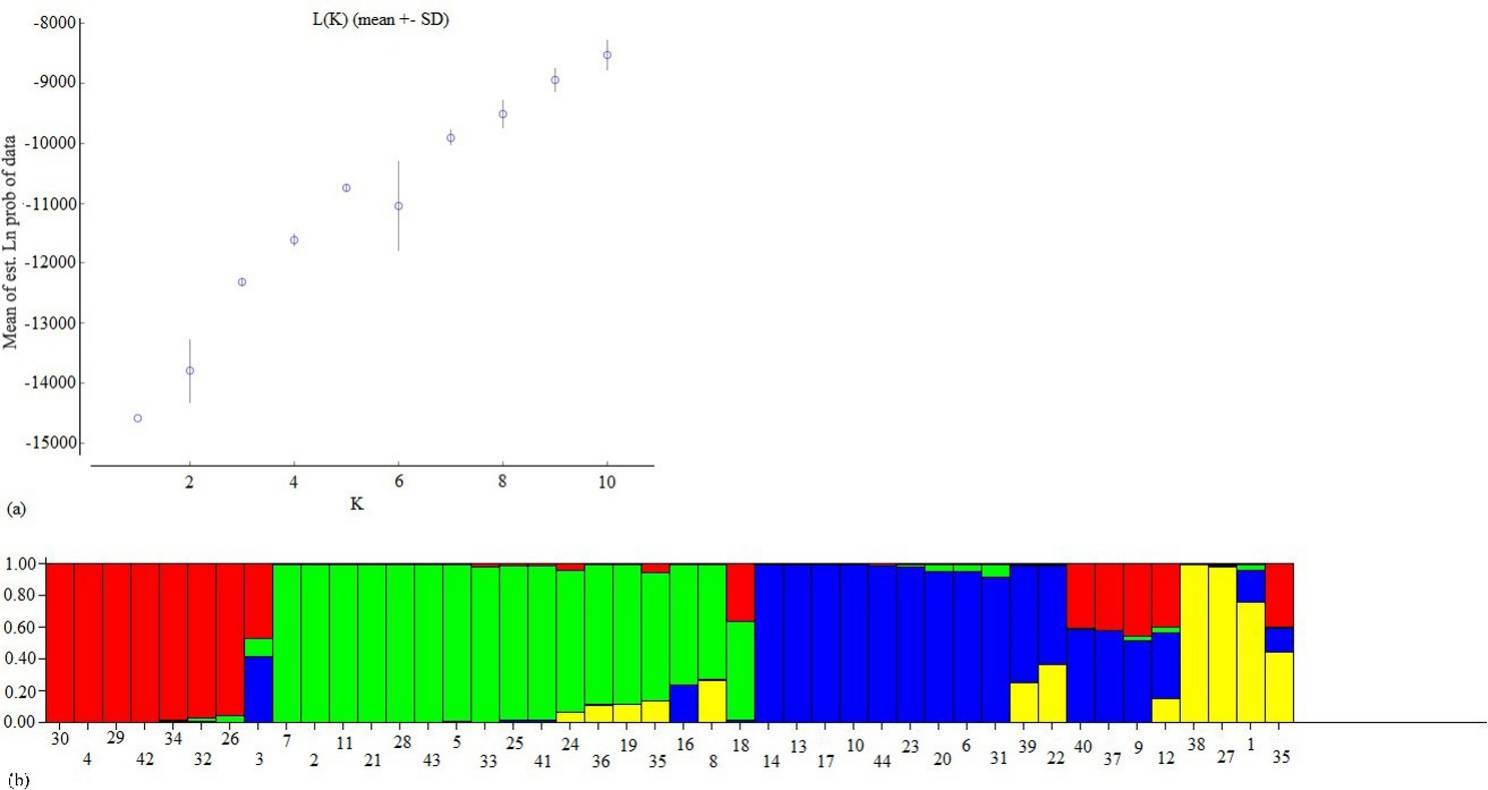
Estimated population structure of 44 pigeonpea genotypes. (a) Average of likelihood value L (K) (mean + SD) against probable number of clusters (K). (b) Barplot of subpopulations at K = 4. Each genotype is represented by a vertical bar and partitioned into colored segments which represent the estimated probability membership in each of the K-inferred clusters on the y-axis. Bar colors (red, green, blue and yellow) indicate the groups identified through the structure program.

The likelihood value method used showed a first clear peak at K = 2 (Fig 2A and S3 Table). However, others larger values of K corresponding to a continuous gradual increase in the likelihood values were observed but the largest was K = 5. This value of K indicated that 5 subpopulations could exist in the analyzed collection. Nevertheless, the best value of K which clearly defined the number of subpopulations was K = 4. As a result, four subpopulations (pop1, pop2, pop3 and pop4) were identified. The subpopulation 1 (in red) grouped 8 genotypes, the subpopulation 2 (in green) grouped 17 genotypes, the subpopulation 3 (in blue) grouped 15 genotypes and the subpopulation 4 (in yellow) grouped 4 genotypes (Fig 2B). 18.18% of admixture supported the 4 subpopulations (S4 Table).

### Genetic diversity

**Fig 3:**
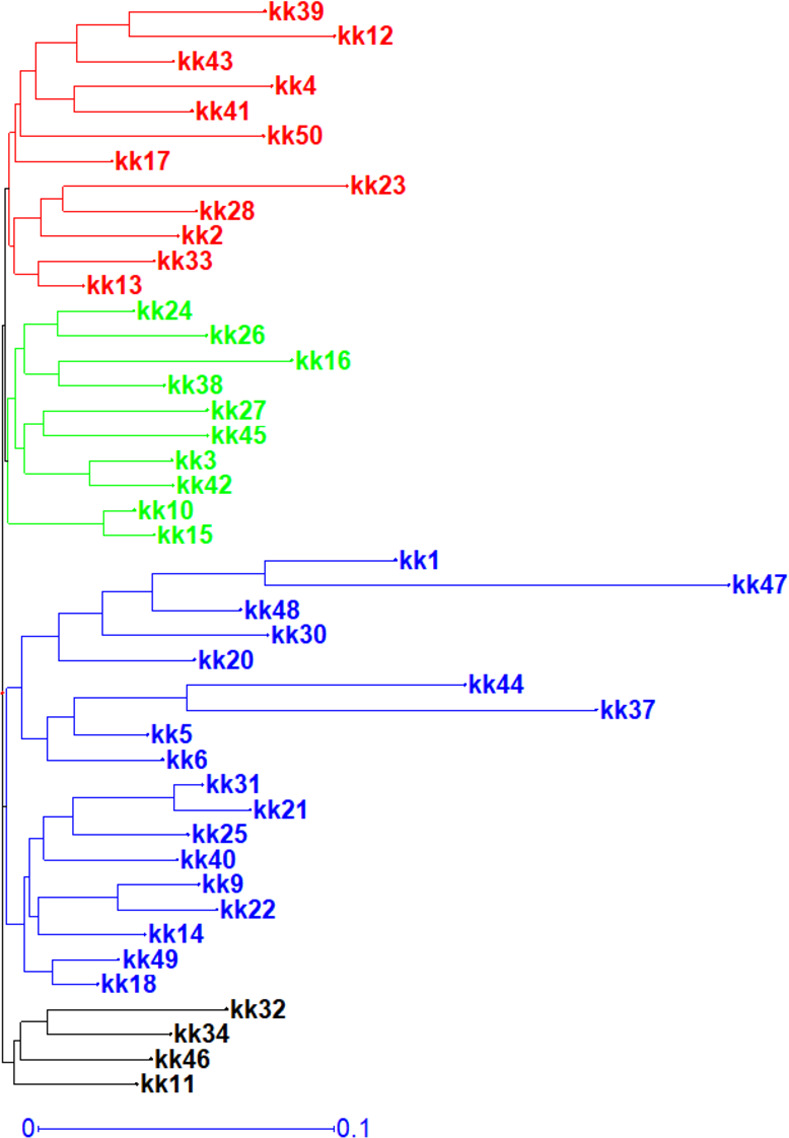
Neighbor—Joining tree of pairwise relatedness among 44 pigeonpea genotypes.

**Fig 4:**
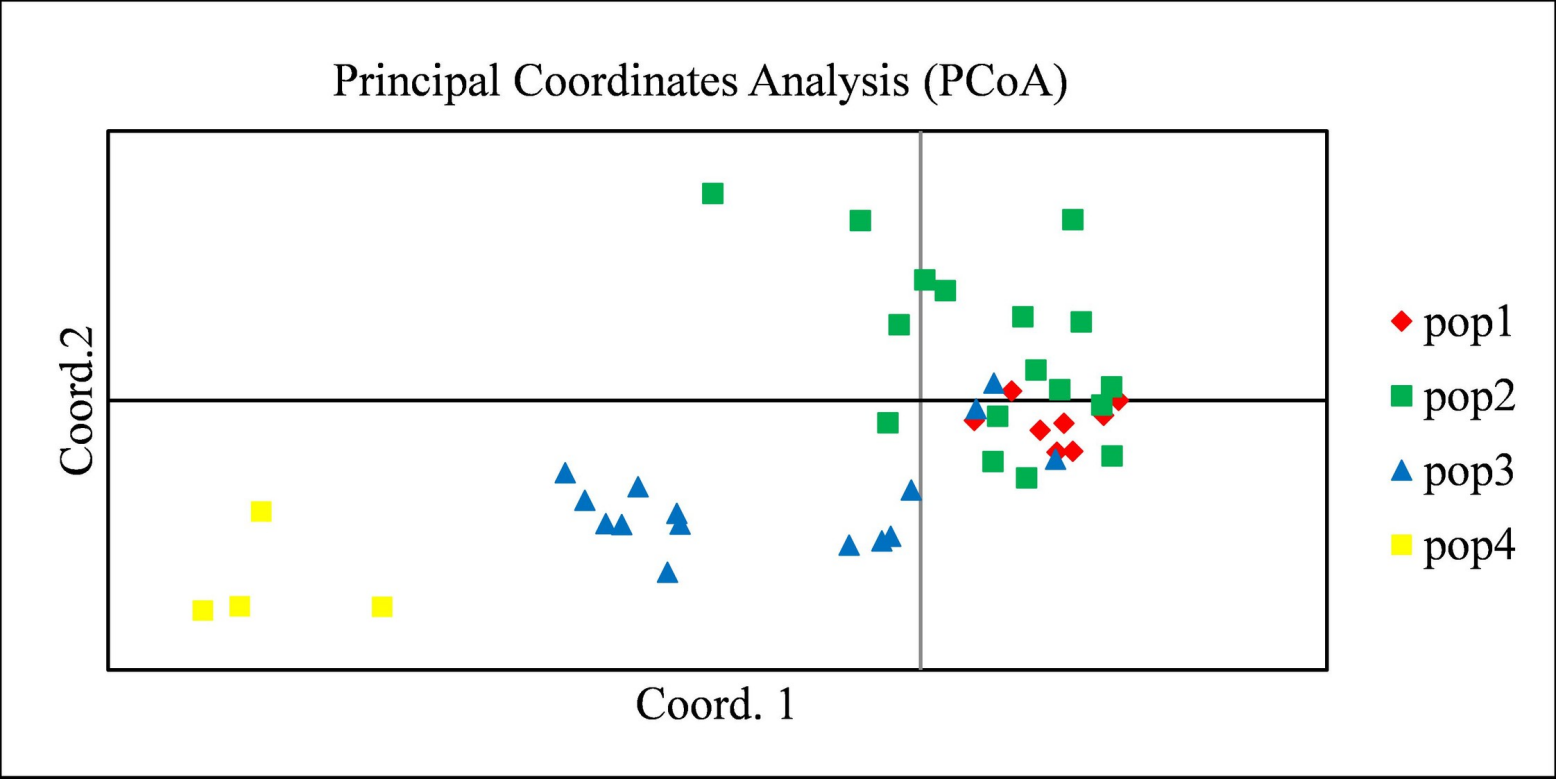
Principal Coordinate Analysis (PCoA) of 44 pigeonpea genotypes based on the population structure result.

The Neighbor Joining tree based on the genetic distances analysis showed four major clusters within all the 44 pigeonpea analyzed genotypes (S5 Table and Fig 3). Cluster 1 (in black) and cluster 2 (in blue) contained 4 and 18 genotypes respectively. Cluster 3 (in green) grouped 10 genotypes and Cluster 4 (in red) grouped 12 genotypes. PCoA was realized with all the 688 SNPs markers. Genotypes were labelled with four different colors according to structure based grouping of genotypes. The first, second and third PC accounted for 33.24% of the cumulative variation. The results did not agree with the structure results but showed an intermixing among genotypes from subpopulation 1, 2 and 3 (Fig 4).

### Analysis of molecular variance and genetic diversity indices

**Fig 5:**
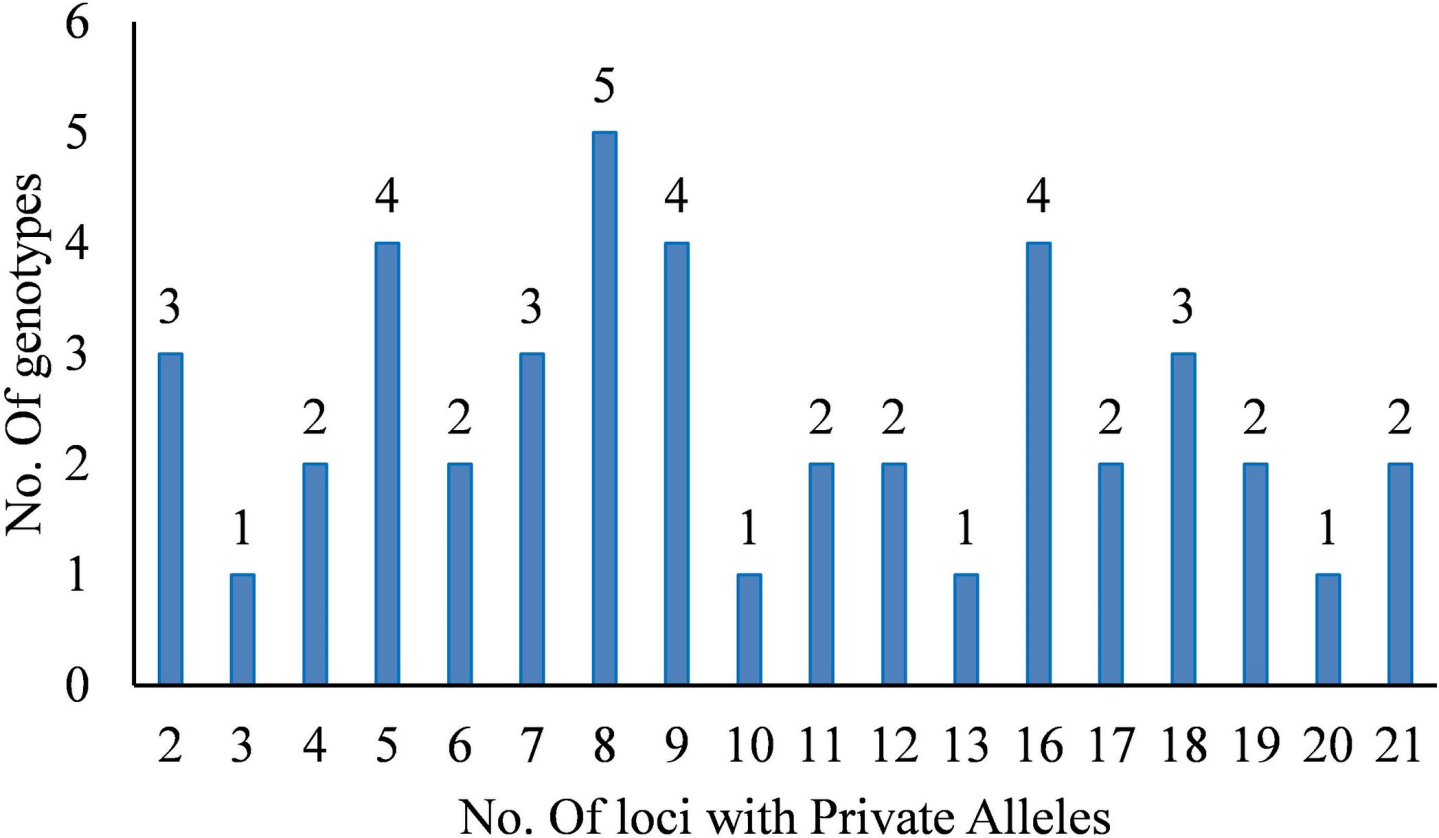
Variation of number of genotypes with private alleles within all the 44 pigeonpea analyzed genotypes.

**Fig 6:**
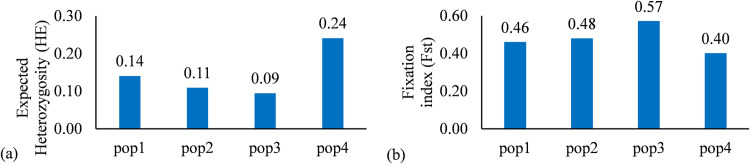
Genetic variation within the subpopulations. (a) Average distances (Expected Heterozygosity) between individuals in same cluster. (b) Genetic variation within the subpopulations (Fst).

The AMOVA results identified 7% of the total variation among subpopulation and 93% was observed within subpopulations. The differentiation genetic index (PhiPT and Nm) were 0.07 and 7.13 respectively (Table 1). The mean value of different alleles (Na) ranged from 1.38 in the subpopulation 1 to 1.57 in the subpopulation 2. The total number of alleles (N) ranged from 4 (in subpopulation 4) to 17 (in subpopulation 2). The percentage of polymorphic loci per population ranged from 37.97% in subpopulation 1 to 57.12% in subpopulation 2. Subpopulation 2 had the highest mean value of loci with private allele (Pa = 0.11) (Table 2). The number of private alleles ranged from 2 to 21 across the genotypes (Fig 5 and S6 Table). The highest Shannon Information index (I) and Shannon diversity index (H) were 0.28 and 0.20 respectively and hold by subpopulation 4 (Table 2). The mean values of *F*st values for subpopulations pop1 pop2 pop3 and pop4 were 0.46; 0.48; 0.57 and 0.40 respectively. We observed a low average distance (*H*_E_) between individuals in same cluster in subpopulation 3 (0.09) (Fig 6).

**Table 1:** Analysis of molecular variance (AMOVA) of the genetic variation among and within the four subpopulations of *C*. *cajan* genotypes using the 688 SNPs.

Source	Df	SS	MS	Est.Var	%
**Among subpopulations**	3	578.89	192.96	7.90	7
**Within subpopulations**	40	4506.12	112.65	112.65	93
**Total**	43	5085.02		120.55	100

*p* value = 0.001. Df, Degrees of freedom; SS, Sum of Squares; MS, Mean Square; Est.Var, Estimated variance; Nm, Number of migrants.

**Table 2:** Variation of different genetic parameters among the four identified subpopulations.

Indices de diversité	pop1	pop2	pop3	pop4
**N**	7.16 (0.03)	14.39 (0.06)	13.13 (0.05)	3.41 (0.03)
**Na**	1.38 (0.02)	1.57 (0.02)	1.50 (0.02)	1.45 (0.02)
**I**	0.19 (0.01)	0.20 (0.01)	0.18 (0.01)	0.28 (0.01)
**Pa**	0.06 (0.01)	0.11 (0.01)	0.08 (0.01)	0.04 (0.01)
**H**	0.12 (0.01)	0.11 (0.01)	0.10 (0.01)	0.20 (0.01)
**P**	37.94%	57.12%	50.44%	44.91%

N, Total number of alleles; Na, Number of different alleles; H, Shannon’s diversity index; I, Shannon’s Information Index; P, Percentage of polymorphic loci; pop, Subpopulation. For each parameter, mean value is used followed by the Standard Error in brackets except the Percentage of polymorphic loci (P).

## Discussion

The knowledge of genetic diversity is of paramount importance not only for the development of conservation strategies but also and more importantly the selection of parents to be used in genetic improvement [46–48]. In this study, we report the second genetic diversity analysis study of pigeonpea using Genotyping-by-sequencing approach in Benin. GBS approach provided 688 high-quality SNPs markers. The mean values of PIC and gene diversity were 0.30 and 0.34 respectively. These values were higher than those reported in Saxena et al. [49] which were 0.17 and 0.2 respectively for PIC and gene diversity across 21 pigeonpea landraces on the one hand and on the other hand 0.19, and 0.24 respectively across 56 pigeonpea elite cultivars. Previously in Benin, mean values of 0.25 and 0.30 for PIC and gene diversity respectively were reported on pigeonpea by using a set of 794 SNPs [37]. The difference between the present diversity index and those reported in Saxena et al. [49] could be explained through the radical certification of certain varieties of pigeonpea. Indeed, in the Indian context where there is selection followed by certification and commercialization of several varieties, the pigeonpea genetic diversity tends towards homogenization by an interesting alleles fixation which leads to the reduction of its diversity [50]. Hence, compared with previous studies, the high level of index of diversity observed in the present study suggested that pigeonpea germplasm encompasses more genetic diversity than reported by previous studies in Benin [51]. Allelic diversity is known for its use as indicator of genetic variation [52]. For the set of 688 SNP markers, we could count 1 376 alleles. This non-negligible total allele’s number is lower than that reported on pigeonpea by Zavinon et al. [37] who reported a total allele’s number of 1 588 using a set of 794 SNP markers generated by GBS approach. For all genetic diversity index calculated, the observed difference with our results and those of previous studies could be explained by the difference between the genetic background of the accessions analyzed or between the number of markers involved in the different studies or the collection size [53–55]. All these findings reinforced the existence of a new structuration in pigeonpea genetic diversity in Benin.

Population structure analysis clarifies genetic diversity studies [8]. This study showed that Beninese’s pigeonpea were divided into four clears subpopulations with a high genetic differentiation between these subpopulations as revealed by a low rate of admixed varieties (18.18%). The Weighted Neighbor Joining tree agreed structure results by giving similar result. The presence of structure in pigeonpea landraces has been reported early in South American by Sousa et al. [56], in Malawi by Njung’e et al. [14] and in Benin by Zavinon et al. [37]. This result supports the findings of Sousa et al. [56] among 77 tested pigeonpea. However, our results contradicted Zavinon et al. [37] who reported three subpopulations with high rate (20%) of admixed genotypes. The presence of a structure within pigeonpea as identified in the present study is of paramount importance. Thus, our results could lay the basis for Genome Wide Association Studies and consequently Marker-Assisted Selection to enhance genetic gain in pigeonpea breeding programs [8]. They could therefore facilitate genes of interest discovery, molecular breeding and allow rapid identification of heterogeneous groups for the development of hybrids with high agronomic performance [57]. Gene flow like selection and genetic drift plays a major role in shaping the genetic structure [58]. In this study, AMOVA results indicated a high level of genetic diversity (93%) within the four subpopulations whereas the rest of the variation (7%) was among subpopulations suggesting a high level of differentiation within subpopulations. The selection for specific agronomic traits by farmers could be the main reason for this high variation within subpopulations. The Nm value (7.13) observed was very high. Knowing that an Nm value less than 1 indicates limited gene flow among subpopulations [59], our result suggested that a high genetic exchange or high gene flow [8] may occur and confirmed that the genetic variation among subpopulations was low when compared to the second level of variation. This result coincided with the PCoA results for which most of the genotypes from subpopulations 1, 2 and 3 showed intermixing among themselves. These findings were in line with those of Kinhoégbè et al. [26] on pigeonpea seed system management in Benin. According to the authors, pigeonpea seed system is informal which favored gene flow through seeds exchanges [26]. They corroborated Radosavljevic et al., [58] who reported that selection and gene flow constituted the main factors of the population structure’s dynamic over time. Hence, these two factors were the main factors that influenced Beninese pigeonpea structure. However, by relying on pigeonpea’s strong tendency to autogamy [60] which is known to contribute to the low genetic variation [61], the low rate (18.18%) of admixed varieties as reported in this study justified the fact that the relatively great diversity obtained in Beninese pigeonpea was due to the selection based on specific traits rather than gene flow.

The diversity pattern revealed by the mean values of total number of alleles, number of loci with private alleles and percentage of polymorphic loci within subpopulations provided insight to genetic diversity within populations [62] indicated a signature in populations [57]. We observed that subpopulation 2 had the highest mean value of the total number of alleles. The loci with private alleles and the percentage of polymorphic loci followed the same trend while the genotypes from subpopulation 4 were the most highly differentiated (I = 0.28; H = 0.20) from those of the remaining populations. This is probably because subpopulation 2 had the largest number of genotypes indicating the high genetic diversity that existed among the genotypes [8]. As a result, the subpopulation 2 represented the main gene pool and could be serve as a source for diverse parents’ selection useful to improve the existing landraces.

## Conclusion

In this study, GBS approach was used to determine the level of genetic diversity, population structure of a collection of pigeonpea landraces grown in Benin. Results showed significant genetic diversity structured and maintained by the selection of cultivars based on specific traits. Four clear subpopulations were identified. The subpopulation 2 exhibited the highest mean values of total number of alleles, number of loci with private alleles and percentage of polymorphic loci. It represented the main gene pool in the analyzed collection and could contain desirable traits, such as biotic or abiotic stress tolerance. These findings laid the basis for Genome Wide Association Studies and consequently Marker-Assisted Selection to enhance genetic gain in pigeonpea breeding programs in Benin.

## Supporting information

S1 Table List of pigeonpea genotypes with corresponding codes and collection sites (District and Village). (XLSX)

S2 Table Genomic distribution of 688 SNPs mapped on the 11 pigeonpea chromosomes. (XLSX)

S3 Table Likelihood value raw summary. (XLSX)

S4 Table Estimated ancestry membership coefficients (Q) for genotypes. (XLSX)

S5 Table Pair-waise genetic distances matrix across 44 pigeonpea genotypes. (XLSX)

S6 Table List of genotypes with one or more private alleles. (XLSX)
