# Expression of Concern: Genetic diversity and population structure of pigeonpea (*Cajanus cajan* [L.] Millspaugh) landraces grown in Benin revealed by Genotyping-By-Sequencing

**DOI:** 10.1371/journal.pone.0350427

**Published:** 2026-06-01

**Authors:** The PLOS One Editors

The *PLOS One* Editors issue this Expression of Concern because we concluded that the article’s [1] peer review was unreliable due to competing interests between the authors, Academic Editor, and reviewers. The competing interests were not disclosed as required by PLOS policy.

This is one of a series of articles for which similar concerns were identified.

The Editors have no evidence of any author involvement in the peer review concerns for this article.

As of the time of this notice PLOS has not reassessed the article's integrity or scientific validity, but our work on this case is ongoing. We regret that the peer review concerns were not identified and addressed before the article was published.
